# Lab and Pilot-Scale Synthesis of M_x_O_m_@SiC Core–Shell Nanoparticles

**DOI:** 10.3390/ma13030649

**Published:** 2020-02-01

**Authors:** Àngela Ribes, Santiago Sánchez-Cabezas, Andy Hernández-Montoto, Luis A. Villaescusa, Elena Aznar, Ramón Martínez-Máñez, M. Dolores Marcos, M. José López-Tendero, Sarai Pradas, Alejandro Cuenca-Bustos

**Affiliations:** 1 Instituto Interuniversitario de Investigación de Reconocimiento Molecular y Desarrollo Tecnológico (IDM) Universitat de València–Universitat Politècnica de València, Camino de Vera s/n, 46022 Valencia, Spain; angelaribes29@gmail.com (À.R.); santiago.sanchez@idm.upv.es (S.S.-C.); anhermo4@doctor.upv.es (A.H.-M.); lvillaes@qim.upv.es (L.A.V.); elazgi@upvnet.upv.es (E.A.); rmaez@qim.upv.es (R.M.-M.); 2 CIBER de Bioingeniería Biomateriales y Nanomedicina (CIBER-BBN), 46022 Valencia, Spain; 3 Departamento de Química, Universitat Politècnica de València, Camino de Vera s/n, 46022 Valencia, Spain; 4 Laurentia Technologies SL.L. Benjamin Franklin 12, 46980 Paterna-Valencia, Spain; mlopezt@laurentia.es (M.J.L.-T.); sarai@topciment.com (S.P.); alejandrocuencabustos@gmail.com (A.C.-B.)

**Keywords:** core–shell nanoparticles, silicon carbide covering, sol–gel synthesis, synthesis up-scaling

## Abstract

The addition of light ceramic particles to bulk technological materials as reinforcement to improve their mechanical properties has attracted increasing interest in the last years. The metal matrix composites obtained using nanoparticles have been reported to exhibit an improvement of their properties due to the decrease in the size of the ceramic additives to the nanoscale. Additionally, important effects such as the dispersion of the nanoparticles, wettability, and low reactivity can be controlled by the modification of the nanoparticles’ surface. In this work, we present the preparation of core–shell M_x_O_m_@SiC nanoparticles with different shell compositions. The accurate and reproducible preparation is developed both at the lab and pilot scale. The synthesis of these core–shell nanoparticles and their scale-up production are fundamental steps for their industrial use as additives in metal matrix composites and alloys. Powder X-ray diffraction and energy dispersive X-ray (EDX) coupled with scanning transmission electron microscopy (STEM) are used to corroborate the formation of the core–shell systems, whereas line scan-EDX analysis allows measuring the average shell thickness.

## 1. Introduction

During the last decades, an increasing amount of interest has been focused on the synthesis and characterization of core–shell particles due to the possibility of adding new functional properties by modification of the particles’ surface [1]. This kind of material consists of individual particles with a core domain covered by another domain that acts as the shell, ranging the composition of any of the two domains from metals, semiconducting oxides, and insulating oxides to polymers. Additionally, great effort has been devoted to the design of nanostructured materials and, as an intersection of these two fields, the design and characterization of core–shell nanoparticles has emerged as a very promising field [2] in which materials with interesting properties such as luminescence [3], selective extraction systems [4], improved thermal conductivity [5], highly selective electrochemical molecular sensitivity [6], dye-sensitizing for solar cells [7], or control release and theranostics [8] have already been reported.

On the other hand, a field that is also progressing rapidly is 3D printing, in which nanoparticles have found promising applications. For instance, polymeric materials have benefited from the addition of carbon nanotubes, graphene, etc. to improve mechanical properties and to lessen weight [9,10]. However, although experiences in adding nanoparticles to metal powder for additive manufacturing (AM) processing do exist, no dispersion optimization is considered, and the industrialization of the process is really poorly defined. Specific problems such as the necessity of high-energy processes to get effective dispersions or a proper wettability of the nanoparticles with the liquid alloy make it sometimes difficult to get the expected mechanical performance of the metal matrix composites (MMCs). Although some studies have already shown the effect of the addition of SiC nanoparticles on the mechanical properties of Ti6Al4V alloy (Ti64) [11], a higher control on the size, shape, and surface of the nanoparticles used as reinforcement is needed. In this scenario, we are particularly interested in the use of nanoparticles for reinforcement applications to improve the mechanical properties of Ti6Al4V alloy. [12]

The synthetic procedures for the preparation of core–shell particles described in the literature are usually related with the hydrolysis of a metallic precursor that forms the shell in the presence of the particles of the core material. Cao et al. [1] reported the synthesis and characterization of core–shell particles in which micrometric SiC particles were covered with different metal oxides (Al_2_O_3_, Y_2_O_3_, and SiO_2_) by means of the corresponding metal salts hydrolysis in the presence of urea. Moreover, other synthetic procedures have been reported for the production of core–shell systems at the nanoscale, based on the acid hydrolysis of alcoxide precursors for the formation of the oxide shell. Thus, researchers have reported on the preparation of TiO_2_@SiC core–shell nanoparticles [13], Pd nanoparticle@TiO_2_ functionalized SiC [6], the simultaneous covering of the SiC nanoparticles by a mixture of iron and titanium oxide [14] by the hydrolysis of the corresponding isopropoxides, and core–shell systems as Al_2_O_3_@MoSi_2_ using aluminum secbutoxide as the precursor [15]. The covering of SiC nanowires with magnetite oxide for the preparation of controllable dielectric–magnetic coaxial hybrid nanowires has also been described [16].

Based on the above, our interest has been the development of a generalized methodology for the preparation of nanoparticles that could allow the fine tuning of the thickness and composition of the shell in core–shell nanoparticles for their applicability in metal matrix composites. Hence, the reported synthetic procedure described herein has allowed us the preparation of M_x_O_m_@SiC core–shell nanoparticles not only at the lab scale but also at the pilot scale, meaning that this up-scaling of the synthetic procedure is an important step toward the industrial fabrication of metal oxide core–shell nanoparticles. The nanoparticles prepared are TiO_2_@SiC, Fe_3_O_4_@SiC, Fe_2_O_3_@SiC, Al_2_O_3_@SiC, and Y_2_O_3_@SiC. The synthesis parameters along with the characterization of the prepared materials are discussed.

## 2. Materials and Methods

### 2.1. Materials

SiC (β-phase, 95%, 50 nm average size) nanoparticles were purchased from Alfa Aesar (Haverhill, MA, USA) or PlasmaChem (Berlin, Germany), depending on the availability of the material in each commercial brand. In the case of the up-scaling process, the SiC nanoparticles were purchased from Hwnano (Guangzhou, China) as they were able to supply a higher amount of nanoparticles. 500 nm SiC nanoparticles (β-phase, 95%, 500 nm average size) were also purchased from Hwnano (Guangzhou, China). In any case, the chosen SiC nanoparticles were those that presented a layer of silicon oxide, as these helped the formation of the oxide shell. The particles covered with a layer of carbon did not allow the formation of the shell. Titanium(IV) isopropoxide (Ti(O^i^Pr)_4_, 97%), aluminum isopropoxide (Al(O^i^Pr)_3_, 98%), aluminum sec-butoxide (Al(O^s^Bu)_3_, 97%), yttrium isopropoxide (Y(O^i^Pr)_3_, solution 20–25 wt.% in toluene), iron(III) acetylacetonate ((Fe(acac)_3_, 97%), and triethylene glycol (TREG) were purchased from Aldrich (St. Louis, MO, USA). Other solvents and acids (hydrochloric acid 37%, glacial acetic acid, 2-propanol, Analytical Grade) were purchased from Scharlab (Barcelona, Spain). All the reactants were used as obtained from the commercial brand without any further purification.

### 2.2. Synthetic Procedure for the Preparation of the TiO_2_@SiC Core–Shell Nanoparticles

The synthesis of the TiO_2_@SiC core–shell nanoparticles is based on the method described by Cernaux et al. [13] and was undertaken by initially dispersing 10 g of SiC (50 nm) in 78 mL of isopropanol at room temperature. In order to diminish the agglomeration of the SiC particles, sonication of the mixture for 15 min was performed. Although some agglomeration of the SiC nanoparticles was still observed after the sonication, no additional dispersant was used to avoid the possible increase in impurities when using these particles in composite materials. Then, 7.8 mL of the titanium precursor Ti(O^i^Pr)_4_ were added dropwise to the previous suspension, and the mixture was maintained with stirring for another 10 min. Once the mixture was properly homogeneous, 7.8 mL of acetic acid and 4.7 mL of distilled water were added to produce the hydrolysis and condensation of the Ti(O^i^Pr)_4_ precursor. The mixture was stirred in air at room temperature, and the solvent was removed by heating under reduced pressure. The resulting dry powder was heat-treated in air at 450 °C for 2 h in a muffle furnace to allow the combustion of the remaining organic material in the titanium precursor. In addition, this heating process allowed completing the dehydration of the primary hydrated oxide and the consequent crystallization of the TiO_2_ as the desired shell onto the SiC nanoparticles.

In order to get a material with a thicker shell without increasing the amount of oxygen in the final core–shell system, we also assayed the same procedure as for TiO_2_@SiC (50 nm SiC), maintaining the weight proportion between the titanium precursor and the SiC, but this time using bigger SiC nanoparticles of an average size of 500 nm.

### 2.3. Synthetic Procedure for the Preparation of the Fe_2_O_3_@SiC Core–Shell Nanoparticles

The synthesis of the Fe_2_O_3_@SiC core–shell nanoparticles was undertaken by firstly dispersing 1 g of SiC (50 nm) in 5 mL of isopropanol at room temperature. In order to diminish the agglomeration of the SiC particles, sonication of the mixture for 15 min was performed. Then, 1.55 g (4.39 mmol) of Fe(III) acetylacetonate (Fe(acac)_3_) was mixed with 20 mL of isopropanol, added to the previous SiC suspension, and stirred for another 10 min. After homogenization of the mixture, 5 mL of acetic acid and 3 mL of distilled water were added to produce the hydrolysis and condensation of the iron precursor. The mixture was stirred in air at room temperature, and the solvent was removed by heating under reduced pressure. The resulting dry powder was heat-treated in air at 450 °C for 2 h in a muffle furnace to allow the combustion of the remaining organic material in the iron precursor. In addition, this heating process allowed completing the dehydration of the primary hydrated oxide and the consequent crystallization of the Fe_2_O_3_ as the desired shell onto the SiC nanoparticles.

### 2.4. Synthetic Procedure for the Preparation of the Al_2_O_3_@SiC Core-Shell Nanoparticles

First, 1.12 g of SiC (50 nm) were weighed into a 250 mL round-bottom flask, and 50 mL of isopropanol were added. This vial was sonicated for 30 min to ensure the homogeneity of the mixture, and then 500 μL of milliQ water were added, after which the mixture was mechanically stirred at 400 rpm. On the other hand, 1 g of aluminum sec-butoxide was weighed into a 100 mL balloon, and then 50 mL of isopropanol were added. This vial was also sonicated to dissolve the aluminum sec-butoxide. The aluminum sec-butoxide suspension was slowly added to the flask containing the SiC nanoparticles suspension and mechanically stirred at 500 rpm for 18 h. After this, the solvent in the mixture was eliminated by heating under reduced pressure, and the residue containing the coated nanoparticles was dried in the oven at 60 °C for another 18 h and finally calcined at 900 °C for 30 min.

### 2.5. Synthetic Procedure for the Preparation of the Y_2_O_3_@SiC Core–Shell Nanoparticles

First, 280 mg of SiC (50 nm) were weighed into a 250 mL round-bottom flask and 75 mL of isopropanol and 15 μL of milliQ water were added. This mixture was sonicated for 30 min in order to ensure the homogeneity, and then it was mechanically stirred. On the other hand, 1 mL of the yttrium isopropoxide toluene solution was introduced in a vial, and then 15 mL of toluene were added drop by drop. This vial was also sonicated until the yttrium isopropoxide was completely suspended. Then, this suspension was slowly added to the SiC-containing flask, increasing the speed of the agitation to favor the mixing of the phases. Finally, the mixture was allowed to stir for 18 h. After this time, the solvent was eliminated by reduced pressure heating, and the nanoparticles were dried in the oven at 60 °C for 18 h. Finally, the powder was calcined in the muffle at 900 °C for 30 min.

### 2.6. Synthetic Procedure for the Preparation of the Fe_3_O_4_@SiC Core–Shell Nanoparticles

The preparation of core–shell nanoparticles in which the metal oxide forming the shell is a mixed valence oxide as is the case of the magnetite Fe_3_O_4_ oxide is more complicated from the procedure point of view. However, we succeeded in the application of the synthesis reported by Liang et al. [16] for the preparation of hybrid noanowires to the synthesis of Fe_3_O_4_@SiC core–shell nanoparticles. First, 10 g of SiC (50 nm) nanoparticles were introduced in a double-neck round-bottom flask, and 300 mL of trietileglycol were added. The mixture was placed for 30 min in an ultrasound bath to properly disperse the nanoparticles. On the other hand, 10 g of Fe(acac)_3_ were placed in a 250 mL flask, 100 mL of TERG were added, and the mixture sonicated for 30 min. This last mixture was added onto the SiC suspension and sonicated for another 30 min. Then, a reflux flask and an argon bubbler were adjusted to the flask, and the mixture was heated at 110 °C under 400 rpm stirring. After that, the heating was increased (2 °C/min) until a temperature of 280 °C was reached and, after 5 min, the mixture was allowed to cool down, maintaining the argon bubbling and the stirring. Once the mixture was cooled down, it was added onto 500 mL of ethyl acetate. The nanoparticles were separated from the solution by centrifugation (9500 rpm, three washing cycles with ethanol) and dried in a muffle furnace at 60 °C during 18 h.

### 2.7. Scale-Up Process for Synthesis of Core–Shell TiO_2_@SiC

**Figure 1:**
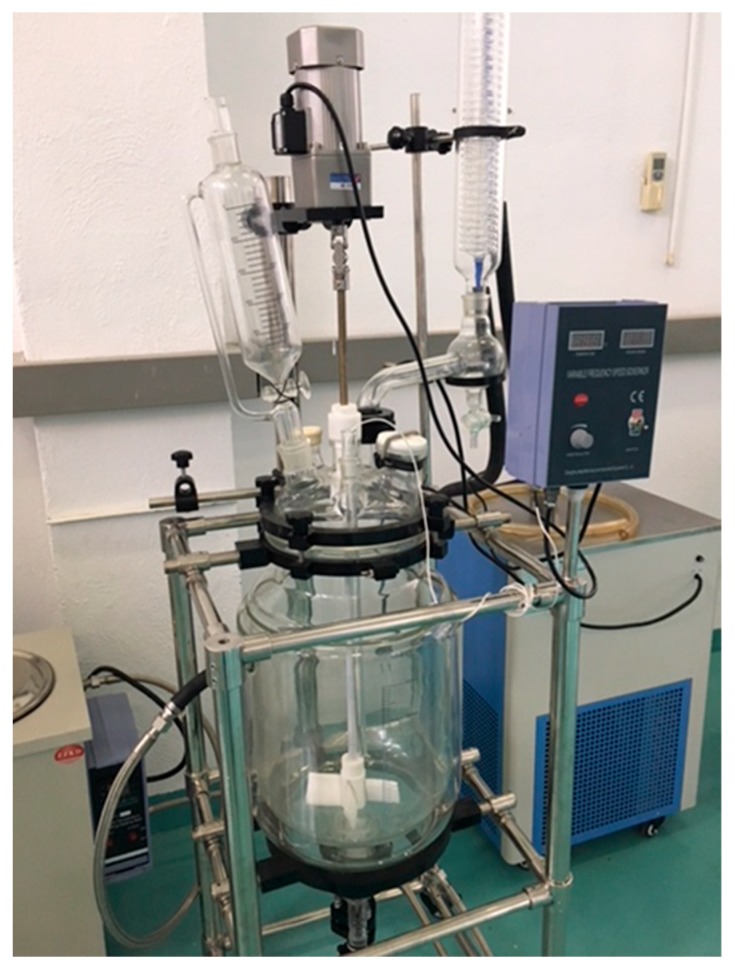
Pilot-scale reactor for the preparation of the M_x_O_m_@SiC core–shell nanomaterials.

Reactor description: The synthesis of core–shell TiO_2_@SiC nanoparticles was performed in a 20 L jacketed glass reactor connected to a heating circulator for further solvent distillation at 90 °C (see Figure 1). The vessel was closed with a cover incorporating five outputs. The central output was allocated for the vertical paddle stirrer. Stirring was accomplished at low medium speed (400 rpm) to get a good heat exchange among the reactants. Another output was connected to a Graham condenser with the purpose of removing the solvent by distillation at 90 °C and recovering it. One output was left for feeding raw materials and the other was used for including an ultrasonic probe (80 kHz). Liquid precursors were incorporated using peristaltic pumps with controllable flow. All the raw materials were weighted under safe conditions into a special cabin equipped with absolute high efficiency particulate air (HEPA) filters. 

Synthesis protocol: In a typical experiment, 934 g of SiC (50 nm) nanoparticles were dispersed in 10 L of isopropanol at room temperature followed by sonication for 15 min to break the particles agglomeration. Then, 1 L of pure Ti(O^i^Pr)_4_ was added with a peristaltic pump (flow 50 mL/min) to the suspension and stirred for another 10 min. Hydrolysis and condensation reactions of Ti(O^i^Pr)_4_ were initiated via the addition of 1 L of citric acid, followed by 600 mL of distilled water. A hydrolysis reaction at room temperature (22 °C) takes 10 min. After the distillation of solvents at 90 °C, some water still remains, forming a slurry of nanoparticles (5% water content) that can be easily recovered from the reactor using a valve situated at the bottom of the container. Then, this slurry was heat-treated to allow the combustion of the remaining organic material in the titanium precursor and also the crystallization of TiO_2_. The heating treatment was carried out at 450 °C for 2 h in a muffle furnace.

The scale-up of TiO_2_@SiC with SiC nanoparticles of 500 nm was performed following the same protocol, but using 210.15 g of starting SiC (500 nm) nanoparticles dispersed in 2.25 mL of isopropanol. In this case, 225 mL of Ti(O^i^Pr)_4_ was added with a peristaltic pump (flow 30 mL/min) to the suspension. Hydrolysis and condensation reactions of Ti(O^i^Pr)_4_ were initiated via the addition of 225 mL of citric acid, followed by 135 mL of distilled water. The distillation of isopropanol, drying, and burning followed the same protocol as that for TiO_2_@SiC (50 nm).

### 2.8. Scale-Up Process for Synthesis of Core–Shell Al_2_O_3_@SiC

The scale-up of core–shell Al_2_O_3_@SiC nanoparticles was carried out in the same reactor configuration described above (Figure 1). First, 28 g of SiC (50 nm) were dispersed in 350 mL of isopropanol at room temperature followed by sonication for 15 min to break the agglomerated particles. Then, 1.4 L of isopropanol were added, which was followed by 28 g of aluminum sec-butoxide and stirring in air at room temperature for 10 min. Then, 35 mL of citric acid followed by 21 mL of distilled water were added. After 1 h stirring, isopropanol was distilled at 90 °C and the remaining water formed a slurry of nanoparticles (5% water content) that was recovered from the reactor using the valve at the bottom of the container. The resulting slurry was dried at 150 °C in an oven and finally burned at 1100 °C for 30 min.

### 2.9. Materials Characterization

Powder X-ray diffraction (PXRD) measurements were taken on a D8 Advance diffractometer using CuKα radiation (Philips, Amsterdam, The Netherlands). Transmission electron microscopy (TEM) images were obtained with a 100 kV CM10 microscope (Philips). High-resolution transmission electron microscopy images (HR-TEM), X-ray emission spectra (EDXS), EDXS element maps, and line scans were acquired in a microscope JEOL JEM-2100F (Tokyo, Japan) operating at 200 kV.

## 3. Results

**Figure 2:**
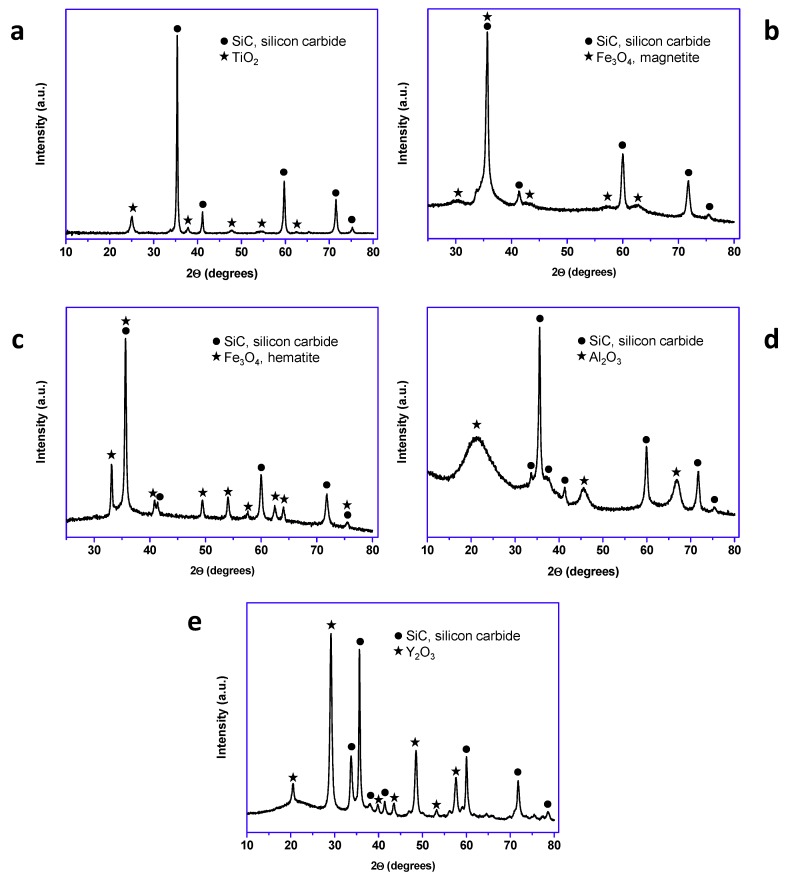
X-ray diffraction patterns of the synthetized core–shell nanomaterials (lab synthesis): (**a**) TiO_2_@SiC; (**b**) Fe_3_O_4_@SiC; (**c**) Fe_2_O_3_@SiC; (**d**) Al_2_O_3_@SiC; and (**e**) Y_2_O_3_@SiC.

**Figure 3:**
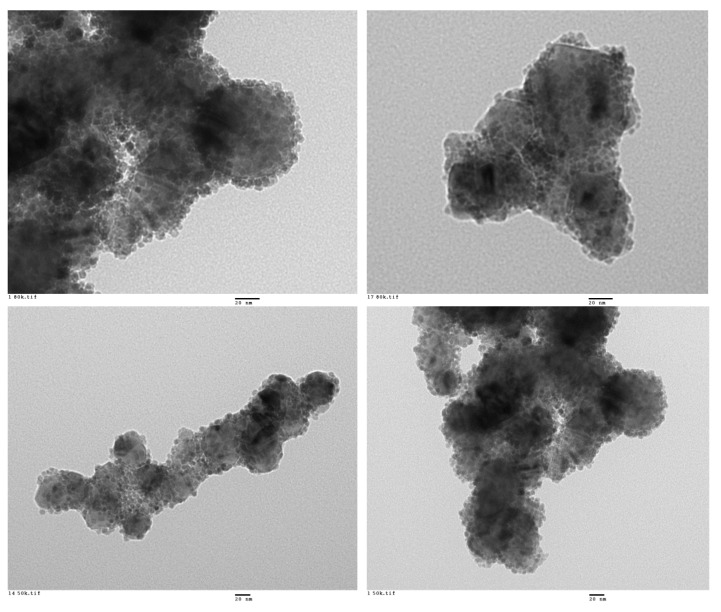
Representative HR-TEM images of the Fe_3_O_4_@SiC core–shell nanoparticles (lab synthesis).

**Figure 4:**
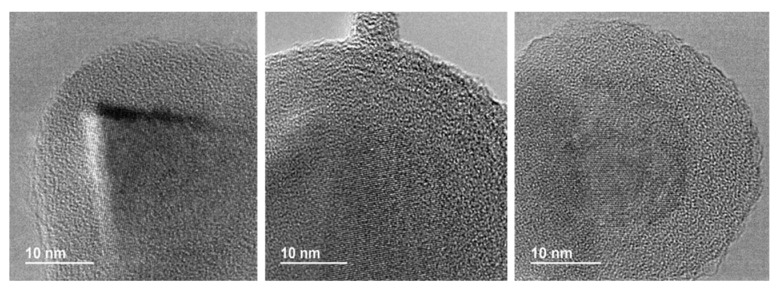
Representative HR-TEM images of the Al_2_O_3_@SiC core–shell nanoparticles (scale-up synthesis).

All the prepared materials have been characterized by powder X-ray diffraction and electron microscopy imaging and EDX analysis. In Figure 2, the X-ray diffraction patterns of the prepared materials are shown. As can be seen, peaks coming from the SiC nanoparticles and peaks from the shell are clearly observed in all cases. A more detailed look into the X-ray diffraction patterns indicated that titanium oxide, hematite, and yttrium oxide present a good crystallinity, while magnetite and aluminum oxide were poorly crystalline. In the case of the magnetite, the low crystallinity can be correlated with the special microstructure of this oxide shell. As can be seen in Figure 3, the Fe_3_O_4_@SiC nanoparticles presented a shell formed by the agglomeration of very tiny nanoparticles of magnetite. However, for aluminum oxide, the formed shell is more continuous (Figure 4), as in the case of the other core–shell particles obtained, and the low crystallinity can be related to the difficulty of aluminum oxide to crystallize at the assayed temperatures. 

**Figure 5:**
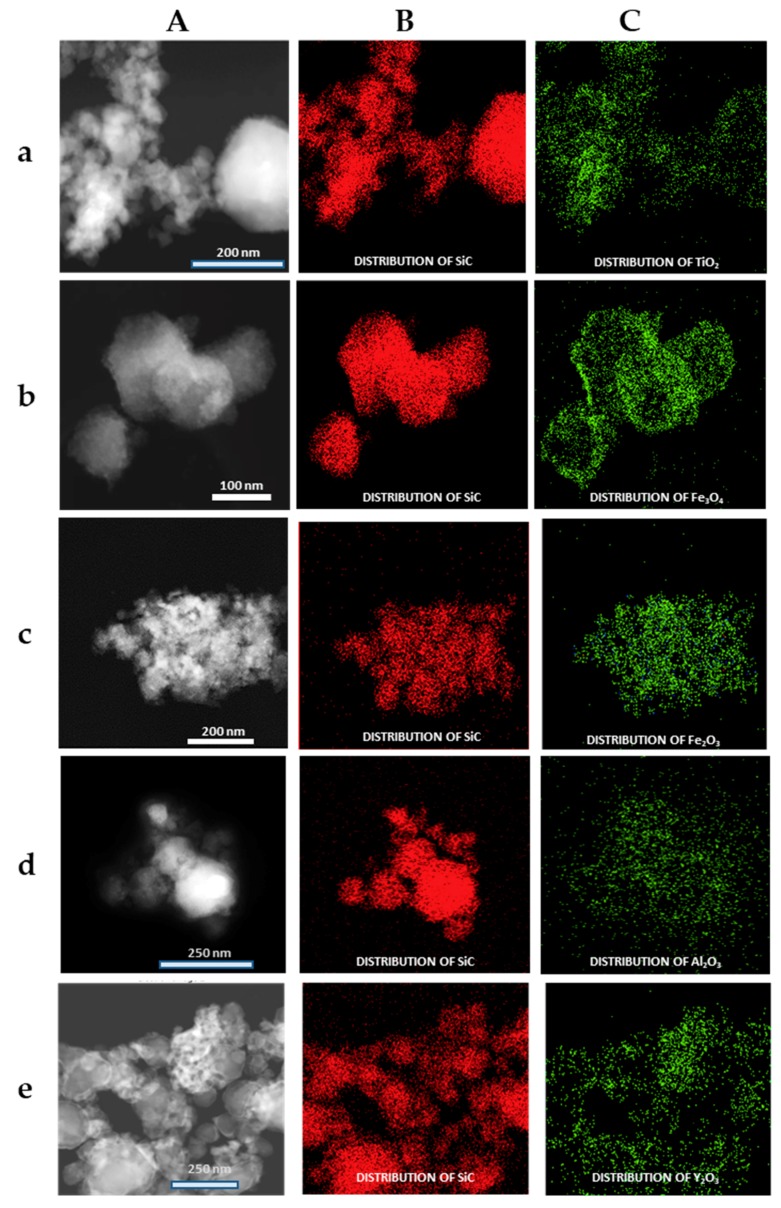
Representative images of the M_x_O_m_@SiC core–shell nanoparticles (NPs) obtained at lab scale (EDX-STEM, mapping tool). (**A**) Direct electron image; (**B**) Signal coming from the SiC particles (Si Kα1 radiation); and (**C**) Signal coming from the shell (corresponding M Κα1 radiation). (**a**) TiO_2_@SiC; (**b**) Fe_3_O_4_@SiC; (**c**) Fe_2_O_3_@SiC; (**d**) Al_2_O_3_@SiC; and (**e**) Y_2_O_3_@SiC. EDX: X-ray emission.

**Figure 6:**
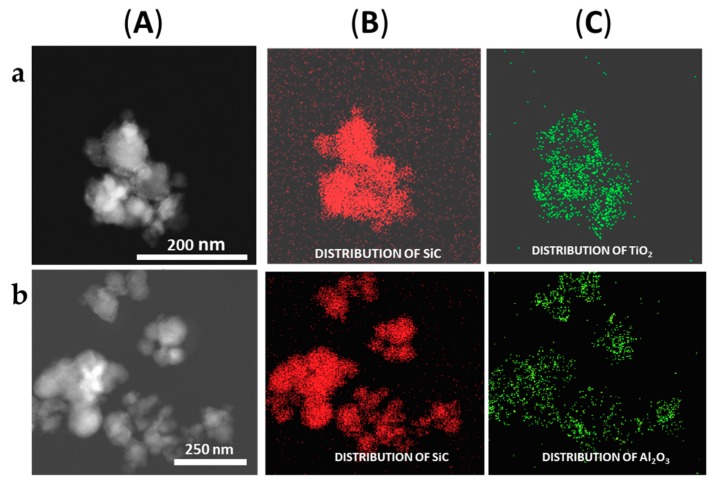
Representative images of the (**a**) TiO_2_@SiC and (**b**) Al_2_O_3_@SiC core–shell NPs obtained in the up-scaling process (STEM-EDX, mapping tool). (**A**) Direct electron image; (**B**) Signal coming from the SiC particles (Si Κα1 radiation); and (**C**) Signal coming from the shell (corresponding MKα1 radiation).

**Figure 7:**
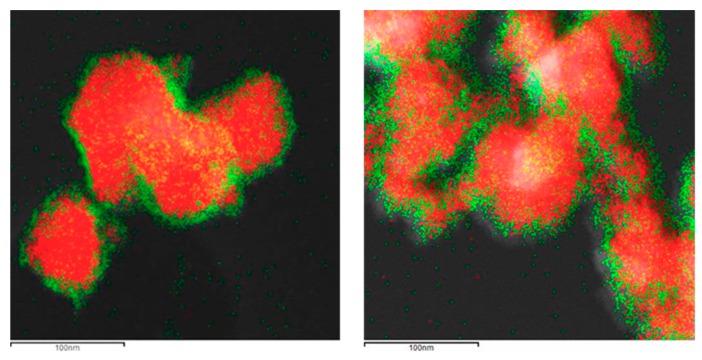
Overlapping of the signals coming from silicon atoms (in SiC, red color as in Figure 5b–B) and that coming from iron atoms (in Fe_3_O_4_, green color as in Figure 5b–C) in Fe_3_O_4_@SiC core–shell NPs (lab synthesis). The thickness of the magnetite shell is revealed.

The formation of the shell in the different nanoparticles was also characterized by the use of the EDX analysis coupled with the STEM microscope using the mapping tool. In Figure 5, representative pictures of the direct electron image (**A** column), the signal coming from the SiC particles (Si Κα1 radiation, **B** column), and the signal coming from the shell (metal Kα1 radiation, **C** column) for the different M_x_O_m_@SiC core–shell NPs materials prepared at lab scale are shown. In every case, the signal coming from the shell colocalizes very well with the one coming from the core, indicating a good shell formation. In the case of the up-scaled materials, a good colocation of the shell and core signals can be observed in Figure 6 also. As an example of the good colocalization of the shell and core EDX maps, the overlapping of both EDX signals is shown in Figure 7 for Fe_3_O_4_@SiC. In these pictures, the homogeneous covering of the SiC core with the magnetite oxide can be clearly seen.

**Figure 8:**
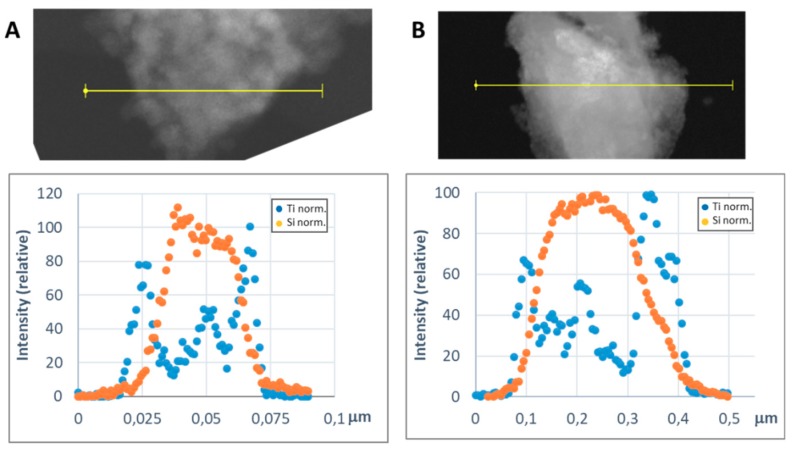
Representative line scans of the M_x_O_m_@SiC core–shell NPs (lab synthesis) (STEM-EDX, line scan tool): (**A**) TiO_2_@SiC (50 nm) and (**B**) TiO_2_@SiC (500 nm). The upper part corresponds to the direct electron image in which the length of the line scan is shown in yellow. In the lower part, the EDX intensity of the Si and Ti elements have been represented along the scan. The intensities have been normalized to the higher value for both the silicon and the titanium signals.

**Figure 9:**
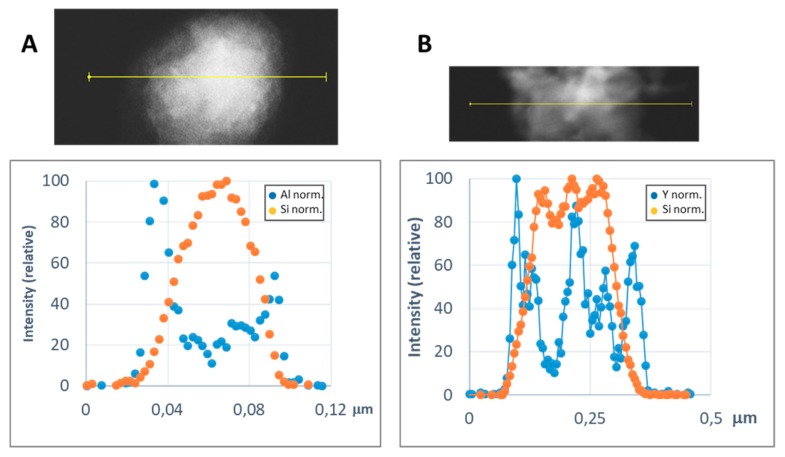
Representative line scans of the M_x_O_m_@SiC core–shell NPs (lab synthesis) (STEM-EDX, line scan tool): (**A**) Al_2_O_3_@SiC and (**B**) Y_2_O_3_@SiC. The upper part corresponds to the direct electron image in which the length of the line scan is shown in yellow. In the lower part, the EDX intensity of the Si and Al/Y elements have been represented along the scan. The intensities have been normalized to the higher value for both the silicon and the metal signals.

In order to get a representative average value of the shell thickness, the EDX line scan tool of the STEM microscope was used. Figure 8 and Figure 9 show representative line scans for some of the prepared materials. In both figures, the direct electron image of the particle is shown with the extension of the scan appearing as a yellow line. Below the electron image, a graphic with the representation of the EDX signal coming from the silicon (core) and the metal of the shell at each point of the line scan is presented. As the amount of material forming the shell is much lower than that of the SiC nanoparticles, in order to level the intensities of the shell and the core to produce these graphics, a normalization relative to the maximum value for each dataset has been applied. In Figure 8, the line-scan data for the TiO_2_@SiC (50 nm) and TiO_2_@SiC (500 nm) can be compared. Figure 9 shows the data for the Al_2_O_3_@SiC and Y_2_O_3_@SiC core–shell nanoparticles. In these graphics, it can be clearly appreciated that the signal coming from the shell starts to increase before the one of the core and that this last one decreases before than the one of the shell oxide, clearly indicating that the oxide is covering the SiC nanoparticles. Therefore, the thickness of the shell has been estimated by measuring the width difference between the signal of the shell and the signal of the core measured at half height of the Si-peak. The obtained values are gathered in Table 1.

## 4. Discussion

The sol–gel methodology has been chosen for the preparation of core–shell nanoparticles, as it allows the homogeneous precipitation of the desired oxides in the presence of the SiC nanoparticles and hence the production of a regular shell. In most of the reported procedures for the preparation of core–shell particles, the amount of material deposited onto the core particles has no special importance, and usually only the perfect covering is pursued. However, in our case, as the nanoparticles are intended to be incorporated in a further work in a Ti64 alloy, the amount of oxide acting as a shell has not only to produce a good coverage but also it must not increase the oxygen content above the Ti64 alloy allowed specifications. The guidelines for the design of the synthetic procedure were to maintain the additional added oxygen as oxide shell to a maximum of a 0.07 wt.% to the total Ti64 alloy when the core–shell nanoparticles were added in a maximum of 1 wt.%. With this in mind, the different proportions of reactants were adjusted, a homogenous shell in only one synthesis step was obtained in every case, and no further oxide precipitation was needed. 

For the preparation of the titanium dioxide shell, slight variations on the method reported by Cernaux et al. [13] based on the direct formation of the oxide onto the SiC nanoparticles by acidic hydrolysis of the titanium precursor (i.e., Ti(IV) isopropoxide) proved to be quite reproducible, and the composition of the core–shell nanoparticles was easy to adjust with the corresponding modification of the reactants proportions. Hence, a shell of TiO_2_ of 5 nm was obtained (Figure 5a), corresponding to a Si:Ti ratio of 7.4. Modifications in the procedure, such as change of the solvent and elimination of the solvents by pressure reduction facilitated the up-scaling synthesis of this nanoparticles (*vide infra*).

In the case of the preparation of the magnetite core–shell nanoparticles, a modification of the procedure reported by Liang et al. [16] based on the so-called polyol method was chosen. This time a metallic complex, iron(III)acetylacetonate (Fe(acac)_3_), was used as a precursor of the metal oxide shell, and a heat treatment in a polyalcohol solvent of high boiling point (TREG) was needed. This synthetic procedure is quite complex, because the final metal oxide must contain both iron(II) and iron(III) cations; then, the synthesis not only needs higher temperature than the one used for the production of the TiO_2_ shell but also working under inert atmosphere. Although the nanoshell obtained presents a nice covering of the SiC nanoparticles (Figure 3, Figure 5b and Figure 7), the complexity of the procedure proved to be not suitable for further up-scaling. 

As the most suitable method to accomplish an up-scale procedure was the one based on the acidic decomposition of the isopropoxide metal precursor, we tried to extend it to the preparation of other core–shell nanoparticles with different shell compositions. The different oxides were chosen not only because of their different properties in relation with covering ability, reactivity, and temperature resistance, but also because of their different oxygen content, which could produce thicker shells maintaining the oxygen content of the final metal matrix composites under the alloy content specifications.

Hence, with some adjustments of the general procedure assayed for the TiO_2_ shell, we obtained core–shell nanoparticles with three other compositions: iron (III) oxide (hematite), aluminum oxide, and yttrium oxide. In the case of the hematite shell, the iron (III) acetylacetonate complex was used as the metallic precursor. For aluminum, the isopropoxide was tried in the first place, but the poor solubility of this precursor produced high inhomogeneity in the formed shells. Hence, the aluminum secbutoxide, which is a liquid at room temperature, was chosen, and a good shell formation was observed. For yttrium, we also found little solubility of the different precursors assayed, and finally a commercial solution of the corresponding isopropoxide was chosen as precursor with good enough results. Comparing the different oxide shells we have prepared for the covering of the 50 nm SiC nanoparticles, TiO_2_ produced a 5 nm shell, whereas Al_2_O_3_ and Y_2_O_3_ produced thicker shells, with 8 nm and 12 nm thickness, respectively.

Once the synthetic process design in the lab proved to be successful in the preparation of core–shell nanoparticles with different compositions, its up-scaling was accomplished. Hence, the preparation of the TiO_2_@SiC core–shell nanoparticles was gradually scaled up by ×3, ×7, ×20 and ×100, and very good results were obtained (Figure 6a). One of the main changes introduced in the scaling-up of the synthetic procedure was the stirring procedure. The intimate mixture of SiC nanoparticles and metal oxide precursor during the hydrolysis–condensation process is a critical parameter in order to get a homogeneous layer of the initial hydrated metal oxide on the SiC nanoparticles. During the lab procedure, a magnetic stirrer was chosen, as the amount of mixture to be stirred was not very large. When moving into a pilot scale, a more efficient stirring procedure was needed in order to maintain the homogeneous mixture of the reactants. Hence, a vertical paddle stirrer was used, and various speed conditions were assayed, of which 400 rpm was the one that gave the best results.

Another important parameter was the addition of the metal oxide precursor. In the lab procedure, the metal oxide precursor solution was added into the SiC nanoparticles suspension manually. However, for the design of the pilot-scale procedure, an automatic method, a peristaltic pump, was chosen in order to get a better control of the metal oxide precursor addition. 

The elimination of solvents in the lab procedure was accomplished by heating the mixture under reduced pressure. However, at the industrial scale, a distillation unit was integrated in the reactor vessel in order to remove solvents in a continuous mode. Additionally, in the lab procedure, the solvents were completely eliminated, while in the scaled procedure, a certain amount of water was left when the distillation of the isopropanol is performed in order to obtain a nanoparticles’ slurry that easily flowed from the reactor bottom outlet and allowed the recovery of the pre-core–shell nanoparticles from the reaction vessel.

Other experimental variables adjusted were (i) the sonication time—tests were carried out varying sonication times from 5 to 90 min; (ii) the continuous addition of metal oxide precursor, which was adjusted through appropriate control of flow with a peristaltic pump, from 2.06 to 10.7 mL/min; and (iii) the hydrolysis time, where different hydrolysis reaction times were assayed from 10 to 30 min. For all these parameters, the effect they have on the shell formation was studied.

Additionally, due to health and safety issues, the replacement of the hydrolysis agent (i.e., acetic acid), was assayed. According to the classification, labelling and packaging (CLP) regulation, glacial acetic acid is classified with the Hazard statements H332 (Harmful if inhaled), H314 (Causes severe skin burns and eye damage), H318 (Causes serious eye damage), and H226 (Flammable liquid and vapor). In the industrial synthesis of the nanoparticles, citric acid (only classified as H319, causes serious eye irritation) was used. 

After the successful adjustment of the reaction conditions for the up-scaled preparation of the TiO_2_@SiC(50nm) core–shell nanoparticles, we tested the validity of the method with the synthesis of other core–shell nanoparticles as Al_2_O_3_@SiC and TiO_2_@SiC (500 nm). The preparation of the aluminum shell was directly accomplished with a 25% scale-up factor by applying the corresponding modifications introduced in the development of the lab procedure (mainly the use of the aluminum secbutoxide precursor) and the assemblage reactor conditions used for the up-scale preparation of the TiO_2_@SiC (50 nm). As it can be seen in Figure 4 and Figure 6b, a coverage similar to the one obtained using the lab-scale procedure was obtained. In the case of the TiO_2_@SiC (500 nm) core–shell nanoparticles, a 20% scale factor was assayed again, obtaining as good results as those of the lab scale preparation.

## 5. Conclusions

In this work, we present the synthesis of M_x_O_m_@SiC core–shell nanoparticles. Starting from the reported procedures, we have been able to produce core–shell nanoparticles in which SiC nanoparticles are covered with an adjustable thickness shell of different compositions. The adjustment of the shell thickness has allowed us to maintain the oxygen content of the core–shell nanoparticles in an optimal value that could permit their use as an additive in MMC as for example with Ti64 (1 wt % of core–shell nanoparticles, 0.07 wt % increase in the oxygen content). Hence, the 50 nm SiC nanoparticles have been covered with a TiO_2_ shell with an adjusted thickness of 5 nm, whereas Al_2_O_3_ and Y_2_O_3_, with a lower oxygen-to-metal ratio, were produced as thicker shells of 8 nm and 12 nm shells, respectively. The use of bigger SiC nanoparticles of 500 nm allowed us to produce an even thicker TiO_2_ shell of 40 nm while maintaining the oxygen content. 

The preparation of TiO_2_@SiC (50 nm), TiO_2_@SiC (500 nm), and Al_2_O_3_@SiC (50 nm) core–shell nanoparticles was successfully scaled up until ×100, ×20, and ×25 pilot scales, respectively. The shell formation was optimized based on different process parameters as the rotation speed of the vertical paddle, flow for the continuous addition of metal oxide precursors, and time allowed for the precursors’ hydrolysis. Finally, pilot processes were adjusted to yield shells with the same homogeneity and thickness as obtained at the lab scale.

## Figures and Tables

**Table 1:** Average shell thickness of the M_x_O_m_@SiC core–shell nanoparticles (lab synthesis) obtained from the EDX line scans analysis.

Core–Shell Particle	Average Thickness of the Shell
TiO_2_@SiC (50 nm)	5 nm
TiO_2_@SiC (500 nm)	40 nm
Al_2_O_3_@SiC (50 nm)	8 nm
Y_2_O_3_@SiC (50 nm)	12 nm
